# Capacitive Measurements of SiO_2_ Films of Different Thicknesses Using a MOSFET-Based SPM Probe

**DOI:** 10.3390/s21124073

**Published:** 2021-06-13

**Authors:** Hoontaek Lee, Kumjae Shin, Wonkyu Moon

**Affiliations:** 1 Department of Mechanical Engineering, Pohang University of Science and Technology (POSTECH), Pohang-si 37673, Gyeongsangbuk-do, Korea; lht1123@postech.ac.kr; 2 Safety System R&D Group, Korea Institute of Industrial Technology (KITECH), 15 Jisiksaneop-ro, Hayang-eup, Gyeongsan-si 38408, Gyeongsangbuk-do, Korea; kjshin@kitech.re.kr

**Keywords:** scanning probe microscopy (SPM), FET sensor, scanning capacitive microscopy (SCM), electrostatic force microscopy (EFM), subsurface imaging

## Abstract

We utilized scanning probe microscopy (SPM) based on a metal-oxide-silicon field-effect transistor (MOSFET) to image interdigitated electrodes covered with oxide films that were several hundred nanometers in thickness. The signal varied depending on the thickness of the silicon dioxide film covering the electrodes. We deposited a 400- or 500-nm-thick silicon dioxide film on each sample electrode. Thick oxide films are difficult to analyze using conventional probes because of their low capacitance. In addition, we evaluated linearity and performed frequency response measurements; the measured frequency response reflected the electrical characteristics of the system, including the MOSFET, conductive tip, and local sample area. Our technique facilitated analysis of the passivation layers of integrated circuits, especially those of the back-end-of-line (BEOL) process, and can be used for subsurface imaging of various dielectric layers.

## 1. Introduction

As semiconductor devices developed, silicon dioxide films became of key importance; these films serve as insulators compatible with silicon devices [1]. Characterization of SiO_2_ films has progressed remarkably. Spatially resolved analysis using scanning probe microscopy (SPM) can be used to determine local electrical properties and microscopic quality. The various SPM techniques include conductive atomic force microscopy (CAFM) [2,3], scanning capacitance microscopy (SCM) [4,5], and electrostatic force microscopy (EFM) [6,7,8,9,10]. All of these methods have been used to analyze SiO_2_ films (usually those of semiconductor devices); in particular, the thick SiO_2_ films of the back-end-of-line (BEOL) process, which is the second step in integrated circuit (IC) fabrication, have attracted much attention [11]. During the BEOL process, interconnections are metallized, dielectric layers for electrical separation are fabricated, and pads and bumps are added; most structures are buried under dielectric films. When evaluating the local properties of such films, spatially resolved measurements derived using SPM are desirable.

In contrast to the ultra-thin oxide films used in the front-end-of-line (FEOL) process, thick films exhibit high electrical impedance, and a highly sensitive SPM technique is required. CAFM [2,3] and SCM [4,5] have been used for many years but can evaluate only dielectrics from several to tens of nanometers thick. Nanoscale capacitance microscopy (NCM) [12,13], which measures the absolute value of capacitance through compensation of the parasitic capacitance, evaluated oxide films up to 100-nm-thick. The theoretical feasibility of evaluating thick oxide films (over 1 µm) using EFM [6,7] and Kelvin probe force microscopy (KPFM) [11,14] has been explored; these techniques measure changes in capacitance over distance (dC/dz values). Recently, scanning microwave microscopy (SMM), involving microwave transmission through a sample, and analysis of the emerging wave has been used to characterize SiO_2_ films hundreds of nanometers to several micrometers thick [15,16]. However, evaluation of thick oxide films remains challenging. It is difficult to obtain absolute values, and SMM requires auxiliary equipment and complex signal processing.

We used a customized “tip-on-gate of field-effect transistor” (ToGoFET) probe to perform the capacitive measurement [17]. The sample electrode was covered with diamond-like carbon (DLC) with lower electrical impedance than SiO_2_ because of leakage. The ToGoFET probe senses the electrical potential of the tip transmitted from the local sample surface. The raw data are amplified by a metal-oxide-silicon field-effect transistor (MOSFET) near the input stage (beneath the tip) for simple signal processing and robustness against parasitic signals. The MOSFET gate exhibits high input impedance, preventing signal loss from the sample to the tip and electrical breakdown. Here, we prepared samples, including several hundred nanometers thick SiO_2_ films, with much higher electrical impedances than the DLC layer studied previously. The measurement results demonstrate the capability of our probe to evaluate the oxide films hundreds of nanometers thick. The feasibility of quantitative measurement was also suggested by showing the change of the ToGoFET signal depending on the oxide film thickness. In discussion, we analyzed the limitations of quantitative evaluation of ToGoFET probe based on our experiments. The development of the ToGoFET probe enables accurate analysis of integrated chip passivation layers, especially those of the BEOL process, and the probe will find many applications in subsurface imaging of dielectric layers.

## 2. The ToGoFET Probe

### 2.1. Basic Principle

**Figure 1:**
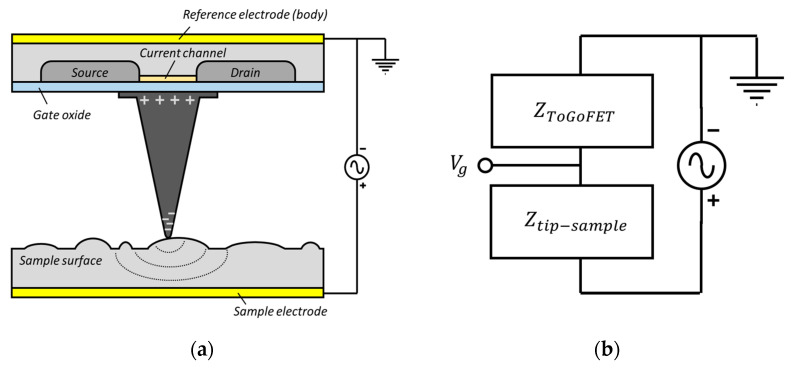
(**a**) Schematic of the ToGoFET probe in contact with the sample surface. (**b**) Equivalent circuit model of the tip-sample system.

The probe featured a conductive tip on the floating gate of a MOSFET (Figure 1).

When the tip contacts a surface, an electrical potential is induced at the tip; this reflects the electrical properties of the local surface, i.e., the surface potential, local capacitance, and trap charge. The gate voltage transmitted from the sample generates an electric field in the channel under the gate; channel conductance is influenced by the induced charge carriers. This principle is expressed by Equation (1):

(1) $$I_{d}=\frac{1}{2}k_{n}{\left(V_{g}-V_{t}\right)}^{2}$$

where $k_{n}$ and $V_{t}$ are a transconductance parameter and MOSFET threshold voltage, respectively. The gate voltage is determined by the ratio of the input impedance of the gate oxide to the impedance of the local sample (Figure 1b). In terms of capacitance, the electrical impedance of a sample increases with the thickness of the oxide layer. Accordingly, the voltage transmitted to the gate decreases, reducing the brightness of the ToGoFET image. As the MOSFET has high electrical impedance, a measurable voltage is transmitted from the sample to the tip. Additionally, the MOSFET renders small signals linear. The probe is robust against signals caused by parasitic (stray) capacitance formed by the electrode of the sample and the electrode on the cantilever structure of the probe. Stray capacitance compromises the ability of SPM to measure electrical properties [18,19]. The robustness of our probe reflected the basic MOSFET principle. The drain current of the MOSFET was saturated above a certain drain voltage, and the MOSFET was thus highly resistant to drain voltage in the region of saturation. In other words, the variation in drain voltage due to the stray capacitance could not have a significant effect on the output signal. As the gate was isolated at the end of the cantilever, with the result that only the drain electrode transmitted a signal through the cantilever, stray capacitance at the cantilever had little influence on the output signal. The high impedance and gain of the MOSFET, and the elimination of stray capacitance (because the gate was isolated), allowed the probe to measure small local capacitances.

### 2.2. Fabrication of the ToGoFET Probe

**Figure 2:**
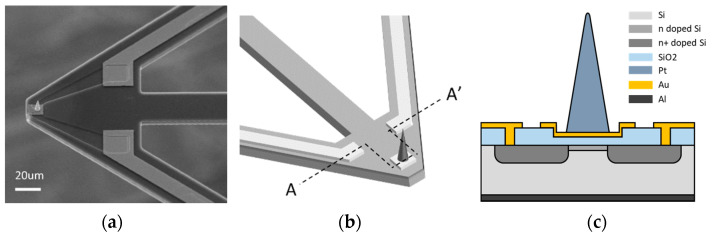
The ToGoFET probe: (**a**) An electron micrograph; (**b**) schematics of the cantilever and built-in MOSFET; (**c**) cross-section of the MOSFET.

We fabricated the probe in three steps: depletion-mode n-type MOS (NMOS) fabrication, cantilever release, and Pt-tip fabrication. Using a silicon-on-insulator (SOI) wafer, we first fabricated a depletion-mode NMOS with a 30-nm-thick gate oxide. To eliminate the need for a sample DC offset voltage, the depletion-mode NMOS had a low dose of ions in the channel region. To release the cantilever structure, a device layer, buried oxide (BOX) layer, and substrate layer were sequentially etched using the DRIE process. Gold was sputtered on the back of the cantilever to reflect laser light. The Pt-tip was deposited via focused ion beam-induced deposition [20]. We laminated the Pt layer as the deposition area was reduced; this created a cone-shaped Pt-tip. The details of this process were published previously [21]. The completed probe is shown in Figure 2.

## 3. Capacitive Imaging of a Buried Electrode

### 3.1. Sample Preparation

**Figure 3:**
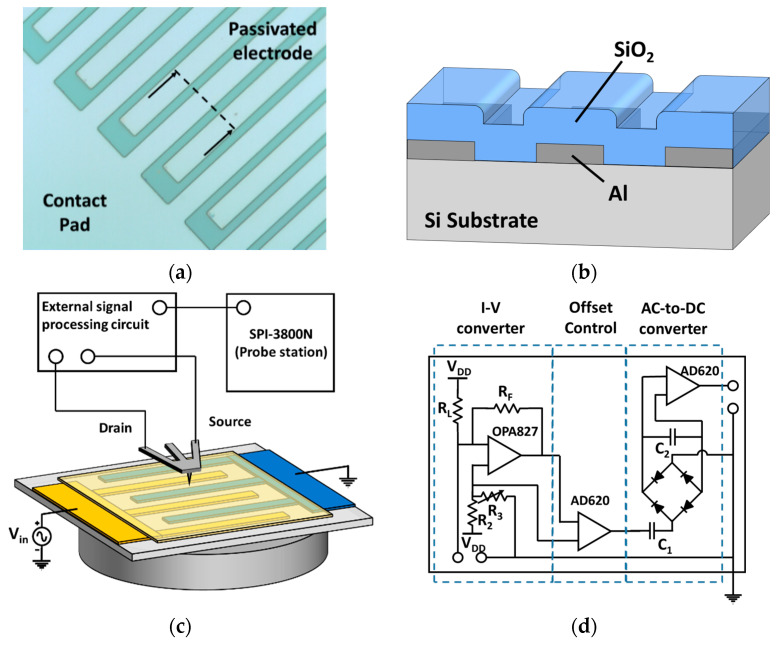
(**a**) Optical micrograph image of the interdigitated electrodes sample covered with SiO_2_ and (**b**) its cross section; (**c**) Sshematic of the measurement setup of the probe; (**d**) the external signal processing circuit, including I-V converter, buffer, and AC-to-DC converter.

Figure 3a,b shows the interdigitated electrode covered with SiO_2_ film. Aluminum was deposited onto a silicon substrate via sputtering (final thickness = 1350 Å) and etched into an interdigitated structure. The electrode lines were 10-µm wide, with 3-µm gaps between interdigitated electrodes. The ends of the electrodes were connected to a pad. After dicing the samples with photoresist passivation, 400- or 500-nm-thick SiO_2_ films were deposited via plasma-enhanced chemical vapor deposition.

### 3.2. Experimental

The measurement set-up is shown in the inset of Figure 3c. The SPM probe operated in contact-mode and an SPA 400 (SPM unit) and SPI 3800 (probe station) were used (Seiko Instruments, Chiba, Japan). The probe was glued at a customized PCB board with gold wire bonding, and jumper wires connected the PCB to the read-out circuit. Topographic images were obtained by the commercial equipment, but the drain current through the probe was processed using the external circuit of Figure 3d, which included an I-V converter, a buffer with offset rejection, and an AC-to-DC converter. Thus, AC drain current was converted to DC output voltage, which entered the user-specified input port. During measurements, sinusoidal voltages of various magnitudes were applied to the sample electrode at 100 kHz.

### 3.3. Local Capacitance Imaging of an SiO_2_-Covered Electrode

**Figure 4:**
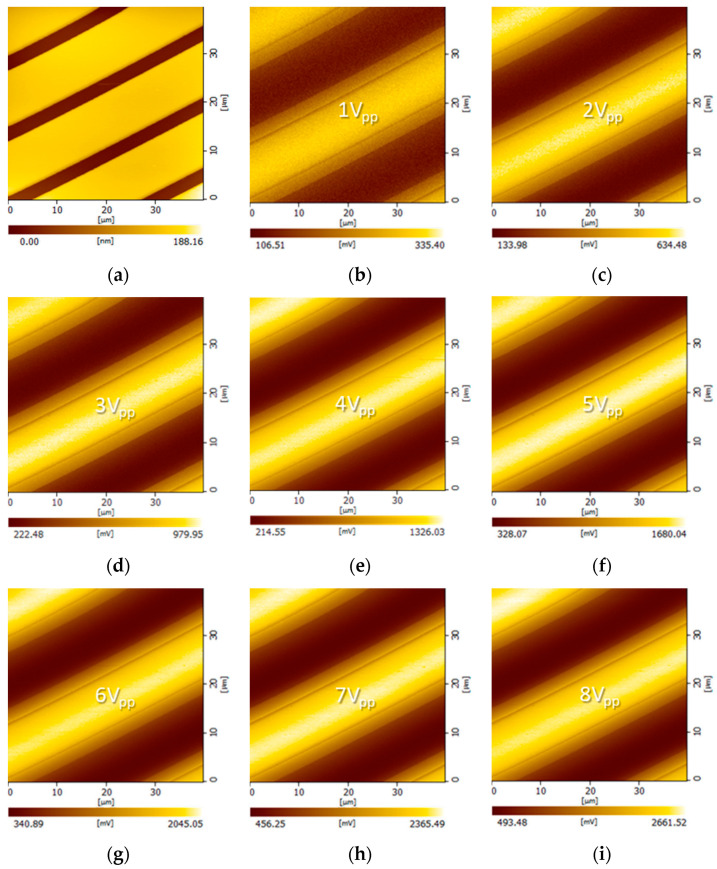
ToGoFET images of interdigitated electrodes covered with 400-nm-thick oxide: (**a**) the topographic image; (**b**–**i**) electrical images were obtained at various voltages with a driving frequency 100 kHz.

Figure 4 shows images of the buried electrode covered with 400-nm-thick oxide, obtained using the ToGoFET probe. Figure 4a shows a topographic image; electrode lines 10 µm in width and with 3-µm gaps can be clearly seen. In the images in Figure 4b–i, the contrast represents the gate voltage transmitted from the electrode.

**Figure 5:**
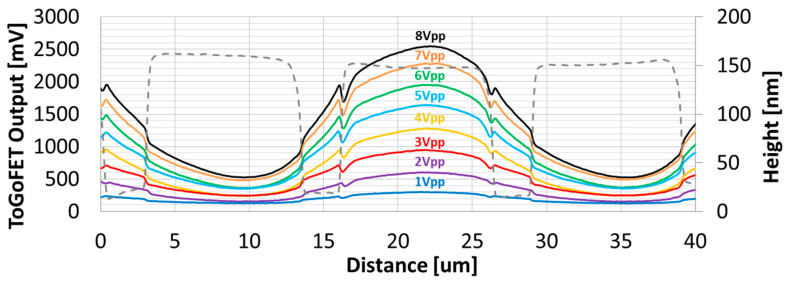
Line profiles of the ToGoFET signal in the direction perpendicular to electrodes covered with 400-nm-thick oxide. The input voltage of each line profile is shown.

In Figure 4b–i, bright regions are biased electrodes, dark regions are grounded electrodes, and medium-brightness regions are the gaps between electrodes. The local capacitance between the tip and sample electrodes varied; the ToGoFET signal thus changed by location, revealing the buried electrodes. It is also possible to measure the voltage transmitted through the dielectric layer; this yields the local capacitance. The ToGoFET signal was linear to 8 V_pp_, as shown clearly in Figure 5 (the line profiles of the ToGoFET signals for the buried electrode). The signals sloped gently and were discontinuous in the gaps between electrodes, reflecting the capacitance created by the conical tip, MOSFET gate, and sample electrodes. The tip-sample capacitance that determines the signal was formed by the sample and entire tip, as opposed to only the tip apex; the value was thus a local average. The discontinuities reflect the fact that the average capacitance of the local area (between the tip and sample) changed discontinuously along the surface structure. A previous study found that the shape of the side wall affected the ToGoFET signal [17].

**Figure 6:**
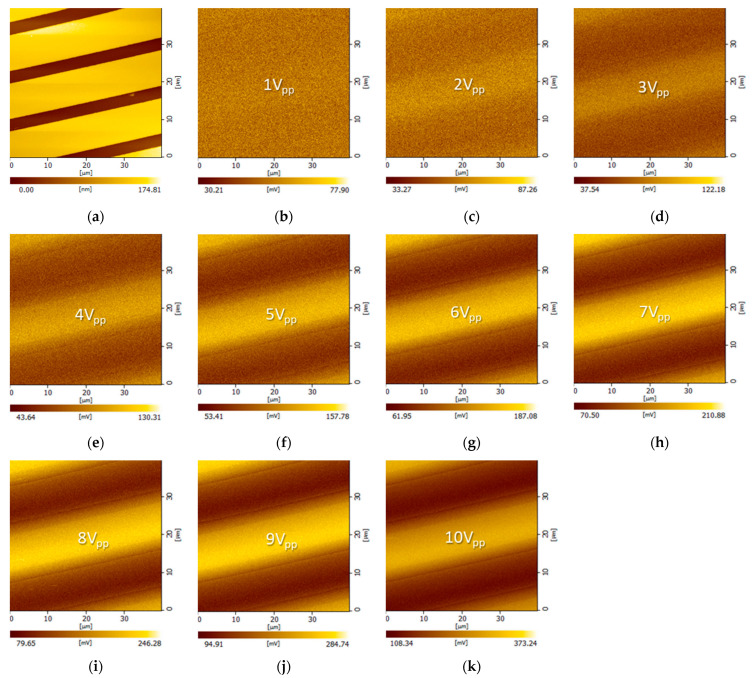
ToGoFET images of interdigitated electrodes covered with 500-nm-thick oxide: (**a**) the topographic image; (**b**–**k**) electrical images were obtained at various voltages with a driving frequency 100 kHz.

In addition, the ToGoFET signal changed depending on the dielectric layer thickness, which in turn affected the local capacitance. Figure 6 shows images of the buried electrodes covered with 500-nm-thick oxide. As the sensitivity was relatively low, measurements were performed up to an input voltage of 10 V_pp_.

**Figure 7:**
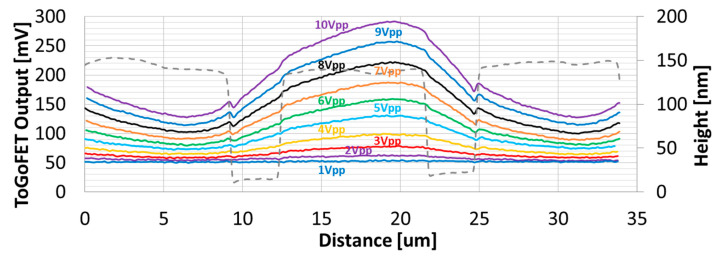
Line profiles of ToGoFET signals in the direction perpendicular to electrodes covered with 500-nm-thick oxide. The input voltage of each line profile is shown.

Compared to the images of electrodes covered with 400-nm-thick oxide, the voltage transmitted from the sample electrode to the tip was lower, and the electrical images were thus affected by inherent noise. Sensitivity depended on dielectric layer thickness, suggesting that the probe can estimate thickness to within a few hundred nanometers. Figure 7 shows more details of the capacitive measurements. No noticeable signal variation along the scan line was evident until 2 V_pp_ was applied, i.e., the sensitivity was low. At a voltage ≥ 3 V_pp_, the probe resolved the electrodes to which the voltage was applied, similar to the 400-nm-thick oxide tests described above. However, averaging became more pronounced as the capacitance between the tip and sample decreased.

## 4. Discussion

**Figure 8:**
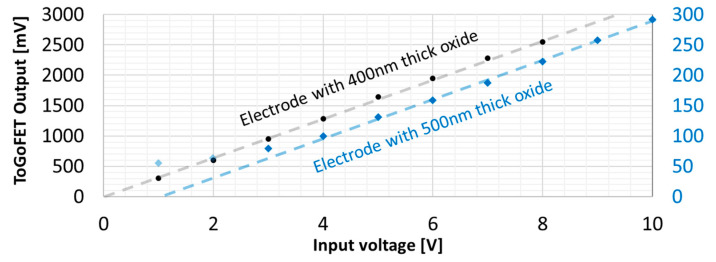
Magnitudes of ToGoFET signals from buried electrodes by input voltage. Noise-dominated data are shown in a light color.

Figure 8 shows the output signals by voltage input to the sample electrode. The data are linear, with the exception of the two noise-dominated datasets shown in Figure 6b,c; these points are shown in a light color. The probe output signal reflects the local capacitance. When imaging buried electrodes, different signals were observed at the same location depending on the oxide thickness. The slopes that exhibited the electrical sensitivities were 321 mV/V and 32 mV/V, respectively, thus they were significantly different from the oxide thickness ratio (4:5). We performed frequency response analysis in an effort to explain the theoretical model of the ToGoFET imaging and this discrepancy.

**Figure 9:**
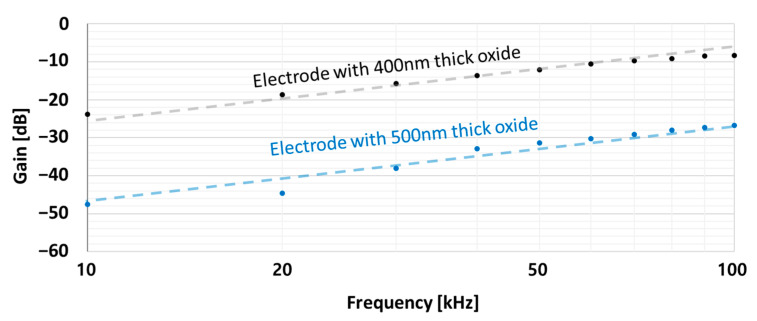
Frequency responses of the ToGoFET probe from 10 kHz to 100 kHz at the center of the buried electrode.

**Figure 10:**
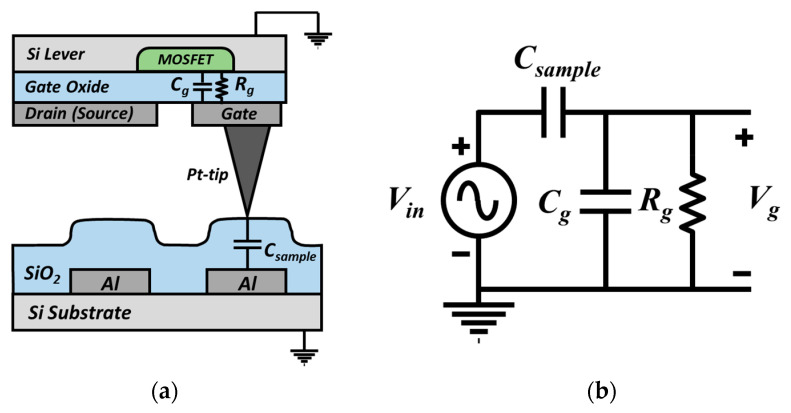
The R-C filter formed between the tip and sample: (**a**) Schematic diagram of the tip-sample structure; (**b**) equivalent circuit model for the structure.

SCM and KPFM both operate within a narrow frequency band when generating a resonance sensor and mechanical vibrations, respectively. The ToGoFET probe can use any frequency within the MOSFET bandwidth. Frequency response analysis was thus possible, and we explored the impedance of the tip-sample system. We placed the tip in the center of the electrode to which voltage was applied. Figure 9 shows the frequency responses from 10 kHz to 100 kHz for the two samples. The trend lines (slopes of 20 dB/decade) are dotted. As described in Figure 1b, the tip-sample system can be interpretated as a series connection of impedances of the gate oxide and sample surface. Silicon dioxide film can be considered as a capacitive component in the circuit model, and when it had a thickness of several hundred nanometers in this study, the resistance component of the film could be neglected. On the other hand, the gate oxide of the ToGoFET probe was much thinner than the sample oxide film and was damaged by exposure to several MEMS processes. Thus, leakage could flow through the gate oxide, which was modeled in parallel to a capacitor and a resistor. Therefore, the measurement system can be described as Figure 10a. In Figure 10b, C_g_, C_sample_, and R_g_ are the gate capacitance, local capacitance between the tip and sample, and gate resistance, respectively; they form a typical high-pass filter. Considering the upward slop at 20 dB/decade, this model agrees with the measurement result in Figure 9 and proves that the output signal from the ToGoFET probe exhibits local capacitance of the sample surface. However, our current probe lacks a gate pad for connection to external equipment. It is difficult to quantitatively evaluate sample characteristics by compensating only for the tip characteristics. Nevertheless, we observed large differences in output signals as the thickness of the oxide film covering the electrode varied. Moreover, the electrical characteristics of the tip sample can be analyzed by deriving the frequency response characteristics.

In future, we will define the electrical impedance of the probe and compensate the images accordingly. It is possible to image and quantitatively evaluate thick oxide films and their subsurfaces, which is challenging using conventional probes. Our probe does not require auxiliary equipment. Signal processing is simple, so it is not difficult to evaluate surface electrical properties, providing better accessibility of the analysis of the dielectric layer using the SPM technique. The probe will find applications in research and industry.

## 5. Conclusions

We demonstrated that our ToGoFET probe can evaluate oxide films hundreds of nanometers thick and provide images of buried electrodes. When the electrodes were covered with 400-nm-thick oxide, the images were clear when the applied voltage was ≥2 V_pp_ and the signals were linear. The image of the subsurface (under the oxide) was clear, so the probe can be used for subsurface analysis. When the electrodes were covered with 500-nm-thick oxide, a higher applied voltage was required to obtain an image. Clearer images were obtained by increasing the voltage to 10 V_pp_. The sensitivity was significantly reduced compared to the 400-nm-thick oxide sample, indicating that a factor other than local capacitance had an effect. Frequency response analysis revealed that gate leakage explained the sensitivity difference. Despite the leakage, the images clearly differed as the oxide thickness varied. As the operating frequency can be chosen at will, subsurface imaging and frequency response analysis of the tip-sample system are possible using only a simple circuit. In future work, our next-generation probe will be subjected to sophisticated frequency response analysis and characterization.
